# Correlation between the Limbus-Insertion Distance of the Lateral Rectus Muscle and Lateral Rectus Recession Surgery in Intermittent Exotropia

**DOI:** 10.1371/journal.pone.0160263

**Published:** 2016-07-27

**Authors:** Ju-Yeun Lee, Eun Jung Lee, Kyung-Ah Park, Sei Yeul Oh

**Affiliations:** Department of Ophthalmology, Samsung Medical Center, Sungkyunkwan University School of Medicine, Seoul, Korea; Bascom Palmer Eye Institute, University of Miami School of Medicine, UNITED STATES

## Abstract

The aim of this study was to investigate whether the limbus-insertion distance (LID) of the lateral rectus (LR) muscle can be a useful indicator for predicting the surgical effect of recession surgery in intermittent exotropia (IXT). Patients who underwent unilateral or bilateral LR recession for the basic type of IXT were included. The distance between the corneal limbus and the posterior edge of the insertion of LR muscle (limbus-insertion distance) was measured intraoperatively using surgical calipers (graded with 0.25 mm precision). We calculated the actual dose-response effect as the difference between the angle of preoperative deviation and the angle of postoperative deviation, and then divided the figure by the total amount of recession at postoperative months 1, 3, and 6. The correlation between the limbus-insertion distance (LID) of LR muscle and each dose-response effect was statistically analyzed. A total of 60 subjects were enrolled in this study. The mean LID of LR muscle was 5.8±0.7 mm. The dose-response effect was 3.2±1.0 prism diopters (PD)/mm at postoperative month 1, 3.4±1.0 PD/mm at postoperative month 3, and 3.4±1.1 PD/mm at postoperative month 6. The LID of the LR muscle was significantly correlated with dose-response effects in cases of unilateral and bilateral LR recession at postoperative months 3 and 6 (*P* = 0.01, <0.01, 0.04 and <0.01 respectively). As the LID of the LR muscle increased by 1 mm, the dose-response effect increased by 0.2PD/mm in unilateral LR recession, and by 0.4 PD/mm in bilateral LR recession at postoperative month 6. In conclusion, the LID of the LR muscle can be used as one predictor of the recession effect to assist in surgical planning for IXT. Moreover, undercorrection at the time of LR recession might be considered in patients with long LID of the LR muscle.

## Introduction

Intermittent exotropia (IXT) is one of the most common types of childhood strabismus in Asia [1]. Although IXT is the most common form of exotropia [2], some aspects of the natural course of IXT remain obscure and exotropic drift frequently occurs after surgery for IXT [3, 4]. It was previously accepted that an initial overcorrection for IXT may result in a better surgical outcome [5]; however, it is challenging to predict postoperative outcomes after recession surgery. Therefore, knowledge of the factors affecting postoperative outcome in IXT is important for surgical planning and patient counseling.

There have been many studies attempting to predict surgical outcomes in patients with IXT. One of the previous studies demonstrated that a large preoperative deviation and a large initial overcorrection are significantly associated with postoperative exodrift [6]. This finding suggested that these two parameters are useful predictive factors of postoperative outcomes in IXT. Another study, however, suggested that the tendon width could have an effect on the outcome of recession surgery in IXT [7].

Although various factors have been considered as predictive indicators, to the best of our knowledge, the limbus-insertion distance (LID) of the extraocular muscle has never been investigated as a predictor of surgical outcomes in patients with IXT. Therefore, this study focused on the LID of the lateral rectus (LR) muscle and the postoperative effect of recession surgery in IXT.

## Methods

This study was a hospital-based, retrospective, observational study of patients who underwent operation by one experienced surgeon (S.O) at Samsung Medical Center between June 2013 and May 2014. The study was approved by the institutional review board of Samsung Medical Center and followed the tenets of the Declaration of Helsinki. Patient records were anonymized and de-identified prior to analysis.

We recruited all patients who underwent primary unilateral or bilateral LR recession for concomitant IXT with normal anterior segment structures. Patients who had eyes with anatomic abnormalities, those who had a history of previous ocular surgery, high myopia (refractive error > -6 D), amblyopia, anisometropia > 2 D, or lateral incomitance (defined as a change of 5 PD or more in the right or left gaze as compared to the primary position), and those in whom the difference between the distance and near angle > 15PD were excluded from this study. All patients underwent complete ophthalmic examinations including prism and alternate cover tests and Titmus stereotesting (Titmus Optical Co, Petersburg, VA, USA). Prism and alternate cover tests were carried out at distances of 6 m in the primary, right, and left gazes and 30 cm in primary gaze.

Under general anesthesia, all patients received unilateral or bilateral LR recession to correct exotropia. The extent of surgery was based on the surgical tables presented in Table 1. During surgery the LR muscle was exposed with a muscle hook after conjunctival incision. After dissecting the muscle from the sclera, the distance between the grey-white line of the corneal limbus and the midpoint of the posterior edge of the LR muscle insertion (limbus-insertion distance, LID) was measured using surgical calipers (graded with 0.25 mm precision). A surgical caliper with a scale grading to 0.25mm was used for measurements. One examiner (J. L) who was blinded to the intraoperative measurements performed all intraoperative measurements.

**Table 1:** Quantum of surgery used for unilateral or bilateral lateral rectus muscle recession in patients with basic-type intermittent exotropia (mm).

Preoperative deviation	Surgery	Dosage
**15PD**	ULRc	8.5
**20PD**	ULRc	9.0
**25PD**	BLRc	6.0
**30PD**	BLRc	7.0
**35PD**	BLRc	7.5
**40PD**	BLRc	8.0
**50PD**	BLRc	9.0

ULRc, unilateral lateral rectus muscle recession; BLRc, bilateral lateral rectus muscle recession

Data on sex, age, age at surgery, age of onset, interval between onset and surgery, preoperative stereoacuity, LID of LR muscle at the time of surgery, preoperative angle of deviation, the angle of deviation at postoperative months 1, 3, and 6 at primary gaze, and axial length were collected through the electronic records. Axial length was measured with an A-scan 1000 ultrasound device (Ophthalmic Technologies International, Toronto, Canada).

We analyzed the correlation between LID of the LR muscle and the dose-response effect for lateral rectus recession. We measured actual dose-response as the difference value between the angle of preoperative deviation in distant primary gaze and the angle of postoperative deviation in distant primary gaze, and then divided the figure by the total amount of recession at postoperative month 1, 3, and 6, respectively.

The correlation between LID of the LR muscle and the dose-response effect was analyzed using correlation analysis. Multiple regression was used to adjust for age, sex, preoperative stereoacuity, preoperative amount of exodeviation, and axial length (SAS Inc., Cary, NC, USA; version 9.4). The preoperative stereoacuity data were categorized and analyzed. (Stereoacuity better than 100 arc/sec was defined as a ‘good’ result.) The difference in the limbus-insertion distance between both eyes was analyzed by intraclass correlation coefficient (ICC). *P* value < 0.05 was considered significant. All data are presented as mean ± standard deviation.

## Results

We enrolled 60 patients (25 males and 35 females) in this study. All patients were diagnosed with comitant intermittent exotropia. The mean age was 9±2 years (range 4 to 15 years), and the mean postoperative follow-up period was 92 days. Twenty one cases of unilateral LR recession and 39 cases of bilateral LR recession were included. The age at surgery was 7±1 years. The interval between onset and surgery was 33±29 months. Demographics of patients enrolled in this study are summarized in Table 2.

**Table 2:** Patient demographics.

Parameters	BLR recession (n = 39)	ULR recession (n = 21)	Total (n = 60)
Sex (M/F)	15/24	10/11	25/35
Age (years)	8±2	9±2	9±2
Age at surgery (years)	7±1	8±2	7±1
Interval between onset and surgery (months)	34±32	30±20	33±29
Spherical equivalent (Diopter)	-1.4±1.8	-1.3±1.0	-1.4±1.4
Axial length (mm)	24±1	24±1	24±1
Preoperative stereoacuity (better than 100arc/sec)	21 (53%)	7 (33%)	28 (47%)
Preoperative exotropia (prism diopter)	27.8±4.5	19.8±1.9	25.0±5.4

BLR, bilateral lateral rectus muscle; ULR, unilateral lateral rectus muscle

The ICC value between both eyes for LID and axial length in bilateral LR recession cases were 0.75 and 0.86, respectively, indicating good agreement between the eyes. We therefore used the mean value of the LID and axial length of both eyes in cases of bilateral LR recession. Overall, the mean LID of the LR muscle was 5.8±0.7 mm (range 4.0 to 7.0 mm, median 6.0 mm), and the mean axial length was 24±1 mm (range 22 to 26 mm). The distributions of the differences in LID and axial length between the eyes are listed in Table 3.

**Table 3:** Differences in the limbus-insertion distance of the lateral rectus muscle and axial length between both eyes of each patient.

Difference (mm)	Limbus-insertion distance (number (%); n = 39)	Axial length (number (%); n = 60)
x = 0.00	10 (25.6%)	3 (5.0%)
0.00<x≤0.25	8 (20.6%)	43 (71.7%)
0.25<x≤0.50	15 (38.5%)	8 (13.4%)
0.50<x≤0.75	2 (5.1%)	1 (1.6%)
0.75<x≤1.00	3 (7.7%)	3 (5.0%)
1.00<x	1 (2.5%)	2 (3.3%)

The mean amount of LR recession was 7.2±1.3 mm (range 5.5 to 9.5 mm, median 7.0 mm). The mean angle of preoperative exodeviation was 24.7±5.6 prism diopters (PD), compared with 1.4±1.8 PD at postoperative month 1, 1.6±1.7 PD at postoperative month 3, and 1.5±1.5 PD at postoperative month 6. The mean dose-response effect in bilateral LR recession was 4.1±0.4 PD/mm at postoperative months 1, 3 and 6. The mean dose-response effect in unilateral LR recession was 2.1±0.2 PD/mm at postoperative months 1, 3 and 6. There were no significant differences in any of these factors between each visits.

Stepwise multiple regression analysis was performed to assess the effect of different parameters. Results revealed that age, sex, stereoacuity, and axial length were not correlated with the LID of the LR muscle or the dose-response effect.

**Fig 1:**
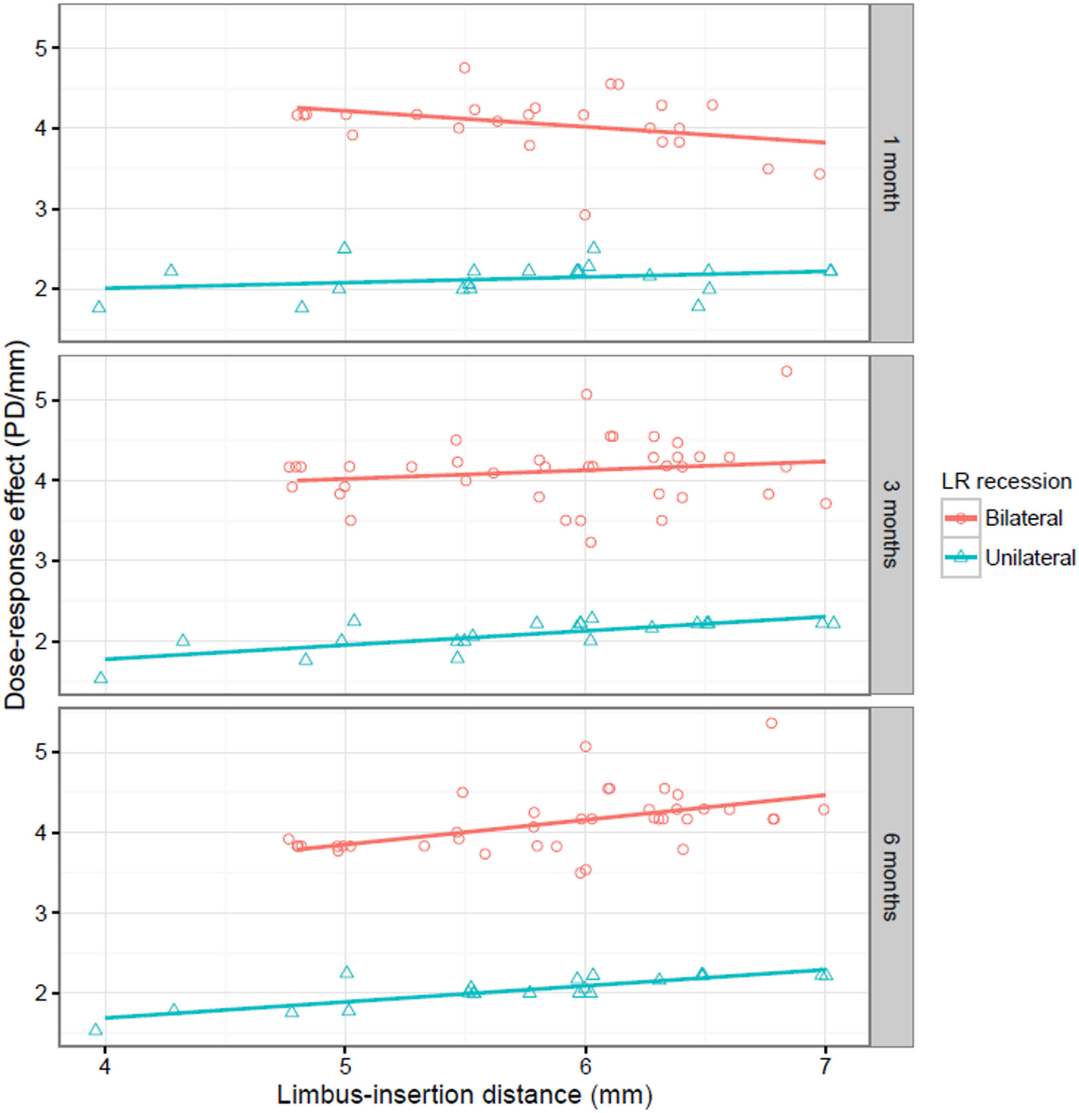
Relationship between the LID of the LR muscle and the dose-response effect of bilateral or unilateral LR recession surgery at postoperative months 1, 3 and 6. (red line: regression line of bilateral LR recession, green line: regression line of unilateral LR recession)

The correlation between the LID of the LR muscle and surgical outcomes including postoperative angle of deviation and dose-response effect is shown in Table 4. There was a significantly positive correlation between the LID of the LR muscle and the dose-response effect at postoperative 3 and 6 months in unilateral and bilateral LR recession cases (*P* = 0.01, <0.01, 0.04 and <0.01, respectively). The LID was negatively correlated with the dose-response effect at postoperative 1 month, but there was no statistical significance in either recession case (p = 0.72 and 0.93, respectively). Fig 1 displays the raw data between the LID of the LR muscle and the dose-response effect at each visit. As the LID of the LR muscle increased by 1 mm, the dose-response effect increased by 0.2 PD/mm in unilateral LR recession, and by 0.4 PD/mm in bilateral LR recession at postoperative month 6. The LID of LR muscle was negatively correlated with the amount of postoperative deviation at postoperative month 6 in both unilateral and bilateral LR recession cases. (all *P*<0.01)

**Table 4:** Correlation between the limbus-insertion distance (LID) of the lateral rectus (LR) muscle and postoperative dose-response effect.

Effect of LR-LID on dose-response effect						
Variable	Unilateral LR recession (n = 21)			Bilateral LR recession (n = 39)		
	Estimate (95% CI)	R-square	*p*	Estimate (95% CI)	R-square	*p*
1 mo	0.1 (-1.1, 0.2)	0.75	0.72	0.1 (-0.3, 0.4)	0.42	0.93
3 mo	0.2 (0.1, 0.2)	0.93	<0.01	0.3 (0.0, 0.5)	0.58	0.04
6 mo	0.2 (0.1, 0.3)	0.91	<0.01	0.4 (0.2, 0.5)	0.66	<0.01

Multiple regression analysis was adjusted for age, sex, preoperative stereoacuity, preoperative amount of exodeviation, and axial length

## Discussion

This study aimed to investigate the relationship between the LID of LR muscle and surgical effect of recession surgery in patients with IXT. We found that the LID of LR muscle was positively correlated with the dose-response effect at postoperative months 3 and 6 in cases of unilateral and bilateral LR recession. This finding suggests that the LID of LR muscle could be considered a predictive factor to assist in planning to correct IXT.

This study was considered as part of a series of previous studies investigating useful indicators to predict surgical outcomes for IXT [6, 7]. Several previous studies reported that various factors, including age at surgery, exotropia type, high AC/A ratio, and lateral incomitance could significantly influence postoperative outcomes [8–11]. The degree of preoperative deviation was also found to strongly influence the postoperative outcomes in IXT [12]. For the sake of accuracy, thus, we only recruited patients with concomitant exotropia without lateral incomitance or significant distance/near differences. In addition, the maximum angle of preoperative deviation in this study was 35 PD, which is less than the value of 60 PD that was reported to skew the postoperative results in previous studies [12]. Therefore, this strategy minimized the effects of other factors that could strongly influence the dose-response effect in uneven conditions.

This study aimed to investigate the dose-response effect according to anatomic factors of the anterior structure in IXT. Lee et al. previously found that the tendon width of the LR muscle was significantly related to the surgical effect of LR recession when the amount of preoperative deviation was less than 25 PD, and suggested that the tendon width of the LR muscle was a useful indicator of the effect of recession surgery in patients with IXT [13]. They demonstrated that the effects of unilateral recession were larger in cases in which the tendon width of the LR muscle was decreased, and the mean effect per millimeter was 3.6, 3.0, and 2.8 PD when ranges of tendon width were 6.5–7.5mm, 8–8.5mm, and 9–9.5mm, respectively [14]. Another group suggested the interpupillary distance was a useful predictor of surgical outcomes in children with IXT [15]. They reported a strongly positive correlation between change in the interpupillary distance and preoperative deviation, and 71% of patients whose changes were outside the 80% confidence interval for expected values had poor surgical outcomes. In this study, we analyzed the LID of the LR muscle as a potential anatomic factor for predicting the surgical effect of recession surgery. In both unilateral and bilateral LR recession, the LID was positively correlated with the dose-response effect after 3 months postoperatively. This finding suggests that the LID of the LR muscle can be used to predict the surgical effect of LR recession in IXT.

The importance of the LID of horizontal rectus muscle and its association with strabismus was reported in previous studies. [16, 17] These studies revealed an abnormal insertion of the medial rectus muscle in patients with congenital esotropia, and considered the LID as one of the factors which influenced the results of surgery related to an equator of the globe. Based on the results of previous studies, the relationship between the LID of the LR muscle and the surgical effect may be explained by the mechanical effect in this study. It is known that posterior fixation of rectus muscle tendons increases the pulley shift, ultimately increasing the effectiveness of transposition [18]. Based on this physiological clarification, it is plausible that excessive recession would move LR muscle insertions into close proximity with pulleys, and this might increase the response of recession surgery. Further studies are needed to verify this relationship.

We also found that the dose-response effect was larger than expected in patients with longer LID of LR muscle. Thus, under-correction is recommended in patients with a LID of the LR muscle that is > 6.0 mm. Based on this result, the adjusted amount of correction in bilateral LR recession is suggested according to the following formula:

$$c=\frac{P}{4.1+0.4\Delta L}$$ (1)

where C is the adjusted amount of correction (mm), P is the preoperative amount of exodeviation (PD), and ΔL is the change in LID of the LR muscle. It can be calculated as ‘(true LID of the LR muscle in patients)–(median value of the LR muscle)’.

In addition, the adjusted amount of correction in unilateral LR recession is as follows:

$$c=\frac{P}{2.1+0.2\Delta L}$$ (2)

For example, there is a patient whose preoperative exotropia is 30 PD. Based on the results of this study, we fixed the median value of the LID of the LR muscle as 6. If his LID of the LR muscle is 4 (shorter than the median value), ΔL will be -2, and the adjusted surgical dose will be 4.1+0.4*(-2) = 3.3 (PD/mm). Hence, when he undergoes bilateral LR recession, we recommend 9 mm of LR recession. Whereas, if his LID of the LR muscle is 8 (longer than the median value), ΔL will be 2, and the adjusted surgical dose will be 4.1+0.4*2 = 4.9 (PD/mm). In this case, we recommend 6 mm of bilateral LR recession. In the same way, when performing unilateral LR recession, the adjusted amount of correction can be calculated using Eq 2 already provided above. These adjustments may be clinically significant to achieve the predicted surgical effect at the time of LR recession. Therefore, we suggest that determining the LID of the LR muscle might help to improve the predictability of recession surgery for IXT and its success rate.

Based on the results of this study, evaluating the LID of the LR muscle before surgery is helpful to determine the amount of recession. Some authors have demonstrated that anterior segment optical coherence tomography (AS-OCT) can accurately detect rectus muscles and shows good agreement with intraoperative measurements [19, 20]. Ngo and colleagues reported a 0.73 ICC value between intraoperative and AS-OCT measurements, with 90% of the measurements within the “clinically acceptable” range with an allowance of ±1 mm between intraoperative and AS-OCT measurements [20]. Hence, it is reasonable to use AS-OCT in detecting the LID of the LR muscle. However, surgeons need to confirm the actual LID intraoperatively, because determination of the adjusted amount of recession should be performed based on precise measurements of the LID of the LR muscle.

This study has several limitations. First, this was a retrospective study with a small sample size and relatively short follow-up period. In addition, all subjects enrolled in this study were children. Therefore, the mean age was skewed, as expected. Most cases of IXT are diagnosed early in childhood. Further studies in patients of all ages with long-term follow up are required in the future. Second, we used a single center for this study and all patients had the same ethnicity, therefore some of the results may not be valid for other ethnic groups. Particularly, the LID of the LR muscle in this study is different from the 6.9mm reported by Fuchs [21]. Differences in ethnicity and age of patients may be responsible for this difference. Therefore, further study in other ethnic groups and other ages is required to verify this theory. At last, we did not investigate the LID of the medial rectus (MR) muscle together with the LR muscle in this study. To more accurately evaluate the surgical effect in IXT, it is necessary to recruit cases of MR resection/LR recession and analyze the LID of MR and LR muscles together. Finally, it can be difficult to calculate the dose-response effect according to the LID of LR muscle because the LID can differ between the eyes. However, most patients in our study had nearly symmetrical distance between the eyes, and there was good agreement in the LID between the eyes of each patient in statistical analysis. Therefore, the differences in the LID of the LR muscle were not considered to affect the dose-response analysis in this study. Regardless, additional well-controlled studies are required to substantiate our findings.

## Supporting Information

S1 Table Limbus-insertion distance of the lateral rectus muscle (LID) in patients with intermittent exotropia. (DOCX)
