# Supplementary material for: Correlation between the Limbus-Insertion Distance of the Lateral Rectus Muscle and Lateral Rectus Recession Surgery in Intermittent Exotropia
Source: PLoS One. 2016 Jul 27;11(7):e0160263. doi: 10.1371/journal.pone.0160263 (PMC4962984; doi:10.1371/journal.pone.0160263)
Supplement: S1 Table — (DOCX) [file pone.0160263.s001.docx]

**S1 Table**. Limbus-insertion distance of the lateral rectus muscle (LID) in patients with intermittent exotropia

| No | Sex | Surgery | Mean LID (mm) | Dose-response effect (PD/mm) |
| --- | --- | --- | --- | --- |
| 1 | F | BLR | 6.5 | 4.3 |
| 2 | M | BLR | 4.8 | 3.9 |
| 3 | M | BLR | 5.0 | 3.8 |
| 4 | F | BLR | 6.4 | 3.8 |
| 5 | F | BLR | 7.0 | 4.3 |
| 6 | M | BLR | 5.0 | 3.8 |
| 7 | F | BLR | 5.0 | 3.8 |
| 8 | F | BLR | 5.9 | 3.8 |
| 9 | F | BLR | 6.0 | 4.2 |
| 10 | F | BLR | 6.0 | 5.1 |
| 11 | F | BLR | 6.3 | 4.2 |
| 12 | F | BLR | 6.4 | 4.2 |
| 13 | F | BLR | 6.6 | 4.3 |
| 14 | F | BLR | 6.8 | 4.2 |
| 15 | M | BLR | 4.8 | 3.8 |
| 16 | M | BLR | 6.0 | 3.5 |
| 17 | F | BLR | 6.8 | 4.2 |
| 18 | M | BLR | 5.3 | 3.8 |
| 19 | F | BLR | 5.8 | 4.1 |
| 20 | M | BLR | 6.0 | 4.2 |
| 21 | F | BLR | 6.1 | 4.6 |
| 22 | F | BLR | 6.3 | 4.2 |
| 23 | F | BLR | 6.4 | 4.3 |
| 24 | F | BLR | 6.4 | 4.5 |
| 25 | M | ULR | 4.3 | 1.8 |
| 26 | M | ULR | 5.0 | 1.8 |
| 27 | F | ULR | 5.5 | 2.0 |
| 28 | F | ULR | 5.8 | 2.0 |
| 29 | F | ULR | 6.0 | 2.2 |
| 30 | M | ULR | 6.0 | 2.0 |
| 31 | M | BLR | 5.0 | 3.8 |
| 32 | F | BLR | 5.5 | 3.9 |
| 33 | F | BLR | 5.5 | 4.0 |
| 34 | M | BLR | 5.6 | 3.7 |
| 35 | M | BLR | 5.8 | 4.3 |
| 36 | F | BLR | 6.0 | 3.5 |
| 37 | F | BLR | 6.1 | 4.6 |
| 38 | F | BLR | 6.3 | 4.6 |
| 39 | F | BLR | 6.3 | 4.2 |
| 40 | F | ULR | 5.5 | 2.1 |
| 41 | M | ULR | 5.5 | 2.0 |
| 42 | F | ULR | 6.0 | 2.0 |
| 43 | F | ULR | 6.0 | 2.1 |
| 44 | M | ULR | 6.0 | 2.2 |
| 45 | F | ULR | 6.3 | 2.2 |
| 46 | F | ULR | 6.5 | 2.2 |
| 47 | F | ULR | 6.5 | 2.2 |
| 48 | F | ULR | 7.0 | 2.2 |
| 49 | M | BLR | 6.3 | 4.3 |
| 50 | M | BLR | 4.8 | 3.8 |
| 51 | M | BLR | 4.8 | 3.8 |
| 52 | F | BLR | 6.8 | 5.4 |
| 53 | M | BLR | 5.5 | 4.5 |
| 54 | M | ULR | 4.8 | 1.8 |
| 55 | M | ULR | 4.0 | 1.5 |
| 56 | M | ULR | 5.0 | 2.3 |
| 57 | M | ULR | 5.5 | 2.0 |
| 58 | F | ULR | 7.0 | 2.2 |
| 59 | M | ULR | 6.5 | 2.2 |
| 60 | M | BLR | 5.8 | 3.8 |
